# Prevalence, Risk Factors, and Antibiogram of Bacteria Isolated from Milk of Goats with Subclinical Mastitis in Thika East Subcounty, Kenya

**DOI:** 10.1155/2018/3801479

**Published:** 2018-11-11

**Authors:** Precious Mahlangu, Naomi Maina, John Kagira

**Affiliations:** ^1^Department of Molecular Biology and Biotechnology, Pan-African University, Institute of Basic Sciences, Technology and Innovation, P.O. Box 62000-00200, Nairobi, Kenya; ^2^Department of Biochemistry, Jomo Kenyatta University of Agriculture and Technology, P.O. Box 62000-00200, Nairobi, Kenya; ^3^Department of Animal Sciences, Jomo Kenyatta University of Agriculture and Technology, P.O. Box 62000-00200, Nairobi, Kenya

## Abstract

A cross-sectional study was carried out to determine the prevalence and risk factors of subclinical mastitis in dairy goats in Thika East Subcounty, Kenya. Further the bacterial pathogens and their antibiogram were investigated. Farm level data on risk factors were obtained from 41 farmers using questionnaires. Milk was obtained from 110 lactating dairy goats and tested for submastitis using California Mastitis Test (CMT). The prevalence of subclinical mastitis at goat level was estimated to be at 50.9% using CMT, out of which 86.5% yielded bacteria on culture. The significant risk factors associated with the occurrence of subclinical mastitis were cleaning schedule (p=0.022, OD=1.047) and parity of the goat (p=0048, OD=1.37). Higher prevalence of subclinical mastitis was observed for goats residing in houses cleaned at least once a fortnight. Does in the first parity were least affected. 169 bacterial isolates were obtained from culture, of which 52 isolates from major classes of isolated bacteria were tested for antibiotic sensitivity to six antibiotics. Fourteen different bacteria were isolated and identified from the milk samples. Coagulase-negative* Staphylococci* (20.7%),* Serratia* spp. (19.5%),* Citrobacter *spp. (16%),* Klebsiella* spp. (11%),* Staphylococcus aureus* (10.7%),* Enterobacter* spp. (6.5%),* Escherichia coli* (5.9%),* Proteus* spp. (3%),* Corynebacterium* spp. (1.8%),* Morganella* spp. (1.8%),* Streptococcus* spp. (1.2%),* Providencia* spp. (0.6%),* Micrococcus* spp. (0.6%), and* Staphylococcus intermedius* (0.6%) were isolated and identified from the samples. All the isolates were resistant to Penicillin G, while 98% of the isolates were sensitive to Streptomycin. In conclusion, the study showed that a large proportion of goats were affected by subclinical mastitis, with the main bacteria being* Staphylococci* spp. and coliforms, and that most of the tested antibiotics can be used in the treatment of mastitis. Farmers need to be trained on improved control of mastitis through adoption of good dairy husbandry and milking practices.

## 1. Introduction

Dairy goat farmers suffer severe economic losses due to intramammary infections [1]. The losses due to mastitis emanate from poor milk quality, reduced milk yield, increased expenditure on treatment, milk discard after treatment, and sometimes death due to the disease itself or through culling of affected livestock [2–4]. Goat milk and its products have a major significance in human nutrition and have been used extensively in feeding starving and malnourished people in developing countries [5]. Consequently, any factor that adversely affects the quantity and quality of milk from the goat is of great economic interest [6]. Although in recent years dairy goat farming has gained interest in central parts of Kenya, challenges such as occurrence of mastitis limit its adoption. Diseases such as mastitis have been shown to hinder the growth of dairy goat production systems [7].

Mastitis is the inflammation of the mammary gland, including not only intramammary tissues but also related anatomical structures such as nipples, mammary areolas, and milk ducts [8]. It is characterized by changes in the physical, chemical, pathological, and bacteriological characteristics of the udder or milk [8–10]. Mastitis leads to milk yield reduction and affects the sensory quality and fatty acid profile of the by-products like cheese [11]. Mastitis manifests either as subclinical, in which there's no visible symptom, or clinical, in which visible symptoms do occur, varying from mild to severe [2]. Subclinical mastitis is also significantly associated with a great increase of leucocytic cells in the milk, which are used as indicators of the condition and milk quality [7]. As subclinical mastitis is less obvious and detectable only by measures of milk's cellular content, it is 15-40 times more prevalent than the clinical form [12]. This is a concern among producers and veterinarians as there are no visible signs of the disease, which can eventually develop into the chronic clinical form of mastitis. Subclinical mastitis in goats is mainly of bacterial origin [13]. Mastitis is generally associated with poor hygienic practices and is caused by the bruising of mammary tissue or teats from traumas, nursing, fly bites, or other wounds to the skin which provide an important barrier to infection [14].

Subclinical mastitis can be recognized indirectly by several diagnostic methods including the California Mastitis Test (CMT), the modified white side test, Somatic Cell Count (SCC), pH, milk electrical conductivity tests, and catalase tests. The SCC and CMT are the most common tests used to diagnose mastitis in dairy goats [15]. The CMT has been found to be more perfect, efficient, and reliable than other field and chemical tests for diagnosis of subclinical mastitis [12, 16–18]. The CMT is based on the reaction between the CMT reagent and the DNA genetic material of the somatic cells. A higher concentration in somatic cells leads to a higher CMT score. CMT scores are directly related to average somatic cell counts [19].

A report on the interpretation of CMT scores on goat milk has been made [20]. It was reported that, in general, milk from noninfected glands will yield a negative (0), trace, or 1 reaction. Scores of 2or 3 are indicative of mastitis. Somatic cell counts in excess of 1,500,000/ml are suggestive of intramammary infection [12].

Some authors have evaluated the sensitivity and specificity of CMT as a rapid, field-based test in the field. In Algeria, the specificity and sensitivity of the CMT were found to be 96% and 99%, respectively [18]. Another study [21] also concluded that CMT can be used as a rapid test as it was found to be highly sensitive in the study.

Correct diagnosis and identification of the etiological agent causing mastitis are essential in determining the treatment strategies [22]. Antibiotics have been used routinely to treat mastitis [23]; therefore an increase in the incidence of mastitis warrants an increase in the use of antibiotics [22]. This increases the risk of antibiotic resistance. There is therefore a need to properly identify the causative pathogen, determine its antibiotic susceptibility profile, assess the most appropriate antibiotic to use, and administer sufficient treatment to completely get rid of the pathogen [23].

In this study, the prevalence, risk factors, and bacterial pathogens causing subclinical mastitis in dairy goats in Thika East Subcounty, Kenya, were isolated and their susceptibility to antibiotics was determined.

## 2. Materials and Methods

### 2.1. Description of Study Area

The study was carried out in Thika East Subcounty of Kiambu County, Kenya. The subcounty is located in the central part of the country, about forty kilometres from Nairobi city. It lies between latitudes 1°S and 1′ south of the Equator and longitudes 37°' and 5′ east. Rainfall is bimodal and ranges from 500 mm to 1,300 mm, while the average temperature is 18.7°C. According to Livestock Production Officers in the study area, farmers have taken up the dairy goat farming, although no proper census has been undertaken (personal communication, Thika East Subcounty Veterinary Officer).

### 2.2. Study Design, Sample Size Determination, and Administration of Questionnaires

A descriptive cross-sectional study design was used. The sample size of 110 lactating dairy goats was calculated using an adjusted formula for small populations [24]. The sampling unit of interest was individual smallholder dairy goat farms whose goat flocks size ranged between 1 and 10. Only farms with lactating goats were visited. Since there was no formal list of dairy goat farmers in the study area, the snowball technique and sampling to redundancy method were used as a sampling strategy to locate the farmers. The initial farmers were identified with the help of the local extension officers. Thereafter, these farmers helped in further identification of other farmers with lactating goats until all the farmers in subcounty were covered. Using this strategy, a total of 41 farmers were identified, from which the lactating goats were identified. At all the farms, details of the lactating goats including age, breed, parity, and lactation stage were obtained from the farmer through administration of a questionnaire.

### 2.3. Milk Sampling and California Mastitis Test

Milk samples were collected aseptically [18]. Briefly, the does were restrained and thereafter the teats were scrubbed with cotton wool saturated with 70% ethanol. The teats were dried using a towel and the first three streams of milk were discarded. 3 ml of milk from separate teats was milked into a CMT paddle and an equal amount of a commercial CMT reagent (Immucell RP, USA) was added to the paddle. The CMT paddle was rotated in a circular motion to thoroughly mix the contents. Gel formation was observed within 20 seconds. The results were read on a score of 0-3. A score of 0, trace, and 1 was considered negative, while a score of 2 and 3 was considered positive. From the CMT positive animals, 5 ml of milk was collected into sterile universal bottles. The samples were placed in cool boxes with ice packs and transported to Microbiology Laboratory, Jomo Kenyatta University of Agriculture and Technology, within 24 hours.

### 2.4. Culture and Identification of Bacteria

The culture and identification were carried out using standard methods [25]. Sheep blood agar (Himedia, India) and MacConkey agar (Oxoid, UK) were prepared according to manufacturer's instructions. The agar was left to set and stored in a refrigerator until use. 100 *µ*l of the milk sample was inoculated onto both sheep blood agar and MacConkey agar. The milk was allowed to dry and streaking was done using a sterile loop. The plates were incubated at 37°C for 24-48 hours.

The morphology of the bacterial colonies obtained was checked for the colony size, shape, texture, and colour. Haemolysis of the red blood cells in the sheep blood agar was also checked for by identification of the changes in the media around and under the colonies. After 48 hours, plates with no growth were recorded as no growth. Plates with mixed growth were subcultured to obtain pure colonies.

The gram-staining technique was used to differentiate between Gram-positive and Gram-negative bacteria [26] and also to note the microscopic shape of the bacteria. Biochemical tests were used to further identify bacteria according to standard methods [25]. Identification of bacteria was primarily made on the basis of colony morphology, haemolytic characteristics, and gram-stain reaction and catalase test. The gram stain was used to differentiate between Gram-positive and Gram-negative isolates. For the Gram-positive rods, the catalase and mannitol test were further used for identification of* Corynebacterium*. The catalase tests, using 3% hydrogen peroxide, were done to differentiate* Streptococcus *from* Staphylococcus* and* Micrococcus. Staphylococcus* and* Micrococcus* species were identified based on their growth characteristics on Mannitol Salt Agar (Himedia, India), slide coagulase test using rabbit plasma, catalase, and oxidase tests (Himedia oxidase disks).* Staphylococcus aureus* was differentiated from coagulase-negative* Staphylococci* using the slide coagulase test.* S. aureus* isolates were further identified using Polymerase Chain Reaction, using* S. aureus*-specific 16S rRNA primers.

The oxidase test was carried out on all Gram-negative isolates to identify the family Enterobacteriaceae. Oxidase-negative isolates were subcultured on MacConkey agar (Oxoid, UK) to determine lactose fermentation. Thereafter, the Triple Sugar Iron (Oxoid, UK) (TSI) test, citrate (Simmon Citrate Agar, Himedia, India), motility (Sim media-Himedia, India), indole (Tryptone broth, Himedia, India), Methyl Red, Voges-Proskauer (Himedia, India), urease (Urea broth base, Himedia, India), mannitol, and catalase tests were used for identification of Gram-negative bacteria. Dichotomous keys [25, 27] were used for identification using results of haemolysis, colony morphology, gram staining, and biochemical tests.

### 2.5. Antibiotic Susceptibility Testing

Antibiotic susceptibility test was performed using the disc diffusion method [28]. Pure cultures of isolates from the seven major isolated bacteria were randomly selected (n=52) and standardized to 0.5 McFarland's standard. Mueller Hinton Agar (Himedia) plates were inoculated with standardized inoculums of the test organism. Six locally available and commonly used antibiotics for treatment of mastitis in Kenya were selected for antibiotic susceptibility testing in the study. The antibiotic discs used were Penicillin G (10 IU), Gentamycin (10*µ*g), Streptomycin (10 *µ*g), Tetracycline (30 *µ*g), Chloramphenicol (30 *µ*g), and Norfloxacin (10 *µ*g). The discs were placed on the agar surface. The plates were incubated at 35°C for 18 hours. The measurement of the inhibition zone was done and results were compared [28]. Results were recorded as resistant or susceptible to specific antibiotics.

### 2.6. Statistical Analysis

The coded data was entered into MS Excel (Microsoft, USA) spreadsheet and exported to SPSS (Microsoft, USA) and R for data analysis. Descriptive statistics were presented as tables. A chi square test was used to evaluate associations between risk factors and mastitis infection (p<0.05). Logistic regression was used to test individual risk factors and their strength of association in mastitis infection. The odds ratio was used to determine the strength of associations identified in the logistic regression procedure.

## 3. Results

### 3.1. Characteristics of Farms and Sampled Goats

A total of 41 farms were sampled from the study area. Most of the farms were a quarter acre (80.5%) and the largest acreage was one and a half acres. The majority of goat houses (75.6%) were raised timbers, while others were earthen. Zero grazing (85.4%) was mostly practiced though some farmers practiced open grazing and tethering. Almost all the farmers (97.6%) kept poultry. Most of the farmers (63.4%) were not aware of occurrence of mastitis in their flocks. The frequency of milking the does ranged from once (80.5%) to twice (19.5%) a day. All the farmers indicated that they did pre- and postmilking teat cleaning procedures. In most of the farms (95.1%), the milk was consumed at home.

### 3.2. Prevalence of Subclinical Mastitis and Identification of the Bacteria

Using the California Mastitis Test (CMT), the prevalence of subclinical mastitis at doe and udder level was found to be 50.9% (56/110) and 40.5% (89/220), respectively. Out of the samples which were positive by CMT, 86.5% (77/89) gave a positive bacterial culture and 169 isolates of bacteria were obtained from the cultures.

The results of the bacteria isolated and their frequency are given in Table 1. In descending order, the isolated bacteria were coagulase-negative* Staphylococci, Serratia *spp.,* Citrobacter* spp.,* Klebsiella* spp.,* Staphylococcus aureus*,* Enterobacter *spp.,* E. coli*,* Proteus* spp.,* Corynebacterium* spp.*, Morganella* spp.,* Streptococcus *spp.,* Providencia *spp.,* Micrococcus* spp., and* Staphylococcus intermedius*.

### 3.3. Relationship between Prevalence and Risk Factors

The results of the CMT were used to evaluate relationship between prevalence and risk factors. In terms of doe breeds, the highest prevalence of subclinical mastitis was found in German alpine (66.7%), followed by crosses, and the least affected breed was the Kenyan alpine. However, there was no significant (p=0.3934, OD=1.059) difference between the breeds and the prevalence of subclinical mastitis [Table 2]. The highest percentage of goats with subclinical mastitis was found in goats aged 4 years and above (57%). However, there was no significant [p=0.2347, OD=1.79] difference between the prevalence of subclinical mastitis and the age of the doe [Table 2].

The highest prevalence (78%) of subclinical mastitis was found in the does in the early lactation stage. However, there was no significant difference (p=0.4251, OD=0.803) between the occurrence of mastitis and the different lactation stages [Table 2].

The lowest prevalence of subclinical mastitis was found in goats in the first parity [Table 3] and a significant (p=0.0477, OD=1.370) increase of prevalence was noted as the parity increased.

The highest prevalence of subclinical mastitis was found in lactating does whose houses were cleaned fortnightly and a significant [p=0.022, OD=1.047] increase was noted with less cleaning [Table 3].

### 3.4. Antibiotic Susceptibility Testing Results

*S. aureus* was most sensitive to Gentamycin (100%), Chloramphenicol (100%), and Streptomycin (100%), followed by Tetracycline (75%) and Norfloxacin (75%). All (100%) coagulase-negative* Staphylococci* (CNS) were sensitive to Gentamycin, Chloramphenicol, and Norfloxacin. Of the CNS isolates, 90% were sensitive to Streptomycin and 80% to Tetracycline. All (100%) the isolates tested were resistant to Penicillin G [Table 4].

Overall, the isolates tested were most sensitive to Streptomycin (98%) and least sensitive to Tetracycline (63%). Of the tested isolates, 31% of the isolates were resistant to Tetracycline and 21% to Chloramphenicol.

Multidrug resistance was found in 2* S. aureus* isolates (resistant to Penicillin G, Tetracycline, and Norfloxacin), 3* Citrobacter* isolates (resistant to Penicillin G, Chloramphenicol, and Tetracycline), 1* Serratia* isolate (resistant to Penicillin G, Chloramphenicol, and Tetracycline), and 2* E.coli* isolates (resistant to Penicillin G, Tetracycline, and Chloramphenicol).

## 4. Discussion

The findings of this work on the prevalence of subclinical mastitis were higher than those reported in Kenya [29], Greece [6], Spain [17], Pakistan [30], and India [31]. The results were comparable to the study in Bulgaria [32] which found prevalence of 44.2%. The results were lower than those reported elsewhere in Kenya and Tanzania [7, 33] which found prevalence of 57% and 77%, respectively. The differences in prevalence of mastitis in various studies have been attributed to the differences in host and management factors which influence intramammary infection of goats [9].

Mastitis has a multifactorial nature with a clear interaction between host, agent, and environment [24]. The differences in prevalence of studies undertaken in Kenya [7, 34] could be due to differences in farm management and climate of the various regions. Further, the high incidence of subclinical mastitis may be attributed to poor hygiene, lack of standard milking procedures, lack of proper pre- and postudder washing, and nonusage of teat dips [7].

The overall prevalence of subclinical mastitis at udder level was lower than that at the doe level. These results are close to those reported by others [6, 7, 17, 35], which reported that udder infection rate was lower than that of doe. In a previous study [7], the left udder had a higher infection rate than the right udder. It has been suggested that this may be due to the fact that the udder which is first milked is more prone to infection than the one that is milked later [7]. This occurs over a long time depending on right or left handedness. As many people in the population are right-handed, there tends to be more infection on the left udder as this is milked first.

The most frequently isolated bacteria pathogen was coagulase-negative* Staphylococci* (CNS). These results are in agreement with other studies [7, 18, 36–39] which concluded that CNS is the causal agent of the majority of mastitis cases in dairy goats. The members of the genus* Staphylococci *are the most important mastitis-causing agents involved in all forms of mastitis even in other ruminants including goats [14].* Staphylococci *are known to cause all forms of mastitis ranging from subclinical, clinical, and acute to gangrenous mastitis [39] and are the major cause of culling in domestic ruminants. This is because* Staphylococci* are detectable on udder skin, inside teat canal, and in mammary glands and are transmitted through improper and unhygienic milking procedures [40].

In the current study, a significant proportion of subclinical mastitis was due to* S. aureus*. Enterotoxin-secreting* S. aureus* intramammary infections are associated with mastitis in dairy ruminants [8]. Subclinical mastitis caused by these bacteria, apart from reducing milk yield, can develop into the clinical form and is the mostly isolated pathogen in clinical mastitis of small ruminants such as goats [13]. The clinical form of mastitis responds poorly to antibiotics because of the development of a tissue barrier that prevents penetration of antibiotics to the site of infection and it is estimated that only 70% of these staphylococcal infections respond to therapy [25]. Further,* S. aureus* secretes thermostable toxins, which enhances the zoonotic role of these pathogens in causing foodborne diseases [38, 40, 41]. Although heating kills the bacteria, the toxins remain in the food, causing food poisoning.

The other commonly isolated bacteria in this study were coliforms which included* Serratia*,* Citrobacter*,* Klebsiella*,* Enterobacter*, and* E. coli*. This is in agreement with other studies [14] which highlighted that coliforms bacteria are the main cause of environmental mastitis in domestic ruminants. Previous studies have also reported that environmental mastitis accounted for over 50% of bacteria isolated from milk of goats having mastitis [42]. Coliforms thrive in unsanitary housing and living conditions of the dairy animals which were highly prevalent in the study area. Some strains of* E. coli* such as the* E. coli* 0157:H7 cause bloody diarrhea in human beings [25].* Citrobacter* has also been shown to cause diarrhea in human beings.

In the present study, the majority of CMT-positive milk samples yielded growth on bacterial culture. High sensitivity (99%) of CMT in diagnosis has been reported [18]. A positive correlation between CMT and the presence of mastitis pathogens in CMT-positive milk samples has also been reported [21]. This means that CMT is a reliable screening tool in the detection of subclinical mastitis and can be used to investigate subclinical mastitis in the dairy goat farms. The test can be used by farmers to screen for subclinical mastitis, since it is a simple, field-based test which is less costly and is easy to be carried out even by the farmers themselves.

In this study, there was significantly higher prevalence of mastitis in does whose houses were cleaned every two weeks compared to those which were cleaned more frequently. These results are in agreement with those by others [7, 13, 18, 33, 38, 43]. Environmental mastitis caused by organisms such as coliforms tends to thrive in dirty environments and hence frequent cleaning of goat houses is recommended.

In the present study, parity was found to be a risk factor for subclinical mastitis. This is in agreement with studies undertaken by others [6, 7, 13, 43, 44]. As CMT results indicated that the prevalence of subclinical mastitis increased with increasing parity, it is recommended to increase screening of does using CMT with increasing parity to curb the development of subclinical mastitis in a dairy herd. Monitoring somatic cell counts on a regular basis and follow-up investigations give an indication of the success of good animal husbandry and hygiene practices. It therefore forms an integral part of mastitis control strategies and assists in diagnosis and treatment [45].

All of the tested bacteria were resistant to Penicillin G. Similar resistance was reported in India [10] and in Brazil [46]. In Pakistan [43], resistance of bacteria to Penicillin G was found to be 57.69%. The current study shows an increased resistance pattern of bacteria to Penicillin G and this is probably due to the long time and extensive use of Penicillin in the treatment of mastitis [10].

Most bacteria in the current study were sensitive to Streptomycin, Gentamycin, and Norfloxacin. A study in Nigeria [47] reported a high resistance of bacterial isolates to Streptomycin. This could have been due to the overuse of the antibiotic in the treatment of goat diseases in the study area. The sensitivity of bacterial isolates in this study to Streptomycin may be because the antibiotic is rarely used in mastitis treatment in the study area. Studies in Bangladesh reported high resistance of* S. aureus* to Streptomycin [48]. In another study in Ethiopia, [49] varying degrees of resistance of bacteria to Chloramphenicol, Gentamycin, and Streptomycin were reported. These studies show that there are differences in sensitivity to antibiotics based on the region and the usage of a particular antibiotic.

Multidrug resistance of bacteria to Tetracycline and Chloramphenicol found in this work is comparable to those [7, 29, 35] which reported multidrug resistance of the bacteria isolated from dairy goats to tetracyclines. The resistance to beta-lactams and tetracyclines is due to the fact that these antibiotics are commonly used in treatment of various bacterial diseases in dairy animals [50]. Results of the current study also found that most bacteria are still susceptible to antibiotics as reported by others [7, 29, 35]; hence, they can still be used in the treatment of subclinical mastitis.

## 5. Conclusion

The current study shows that dairy goats in Thika East Subcounty of Kenya had high prevalence of subclinical mastitis. Since farmers are not wary of the occurrence of the subclinical disease, they could be having major economic losses through reduction in milk yield. Farmers in the study area should be encouraged to use California Mastitis Test to screen for subclinical mastitis as it is highly sensitive and is affordable. The genus* Staphylococci* was the main etiological agent of contagious subclinical mastitis. The study showed that hygiene plays a big role in the occurrence of subclinical mastitis; hence, the farmers should ensure good sanitation and maintain strict cleaning schedules for goat houses to reduce the occurrence of subclinical mastitis. Most broad-spectrum antibiotics are still effective in treating subclinical mastitis. However, Penicillin G should not be used in the treatment of subclinical mastitis. The extension workers in the area should train farmers on improved animal husbandry practices such as good housing, maintenance of regular cleaning schedule, and proper milking procedures.

## Figures and Tables

**Table 1:** Bacteria isolated from dairy goats in Thika East Subcounty, Kenya.

Bacterial species	Number of isolates	Percentage (%)
Coagulase-negative *Staphylococci*	35	20.70
*Serratia* spp.	33	19.53
*Citrobacter* spp.	27	15.98
*Klebsiella* spp.	19	11.24
*Staphylococcus aureus*	18	10.65
*Enterobacter* spp.	11	6.51
*E. coli*	10	5.92
*Proteus* spp.	5	2.96
*Corynebacterium* spp.	3	1.78
*Morganella* spp.	3	1.78
*Streptococcus* spp.	2	1.18
*Providencia* spp.	1	0.59
*Micrococcus* spp.	1	0.59
*Staphylococcus intermedius*	1	0.59

Total	169	100

**Table 2:** Effect of breed, age, and lactation stage on prevalence of subclinical mastitis in dairy goats in Thika East Subcounty as identified by CMT.

**Risk factor**	**Proportion positive**	**Prevalence of mastitis (**%**)**
**Breed **		
German alpine	6/9	66.7
Crosses	26/46	56.5
Toggenburg	10/20	50
Others	5/10	50
Kenyan alpine	9/25	36
**Age (yrs)**		
1	2/3	67
2	18/39	46
3	19/38	50
4 and above	17/30	57
**Lactation stage**		
Early	14/18	78
Mid	16/39	41
Late	26/53	49

Early: 1 day-3 months, mid: 3-6 months, and late: >6 months.

**Table 3:** Effect of parity and cleaning schedule on prevalence of subclinical mastitis in dairy goats in Thika East Subcounty as identified by CMT.

**Risk factor**	**Number of positive does**	**Proportion (**%**)**
**Parity**		
1	15	43
2	22	48
3	7	58
4 and above	12	71
**Cleaning schedule**		
Weekly	30	46
Fortnightly	19	73
Daily	4	33
Irregular	3	42

**Table 4:** Resistance pattern of bacteria isolated from milk of goats with subclinical mastitis to 6 antibiotics.

	**Percentage (**%**) of resistance**					
**Bacteria**	**Chloramphenicol**	**Penicillin G**	**Tetracycline**	**Norfloxacin**	**Streptomycin**	**Gentamycin**
*S. aureus (n=8)*	0	100	25	25	0	0
CN*S (n=10)*	0	100	20	0	10	0
*Serratia (n=9)*	33	100	22	0	0	0
*Klebsiella (n=8) *	25	100	37	0	0	25
*Citrobacter (n=7)*	57	100	57	0	0	0
*Enterobacter (n=6)*	0	100	17	0	0	0
*E. coli (n=4)*	50	100	0	0	0	0

n = 52.

## Data Availability

The raw data on prevalence of subclinical mastitis, risk factors, and antibiotic sensitivity used to support the findings of this study are available from the corresponding author upon request.
